# Supplementary material for: Utilizing Molecular Simulations to Examine Nanosuspension Stability
Source: Pharmaceutics. 2023 Dec 28;16(1):0. doi: 10.3390/pharmaceutics16010050 (PMC11154398; doi:10.3390/pharmaceutics16010050)
Supplement: Supplementary file 1 [file pharmaceutics-16-00050-s001.zip › pharmaceutics-2732314-supplementary.pdf]

Supporting Information: Utilizing Molecular Simulations to Examine Nanosuspension Stability

Andrew P. Latham,[*,†,‡,¶] Elizabeth S. Levy,[†] Benjamin D. Sellers,[‡] and Dennis H. Leung[*,†]

†*Small Molecule Pharmaceutical Sciences, Genentech, Inc., 1 DNA Way, South San Francisco, CA 94080 USA*

‡*Discovery Chemistry, Genentech, Inc., 1 DNA Way, South San Francisco, CA 94080 USA*

¶*Current address: Department of Bioengineering and Therapeutic Sciences, Quantitative Biosciences Institute, University of California, San Francisco, San Francisco, CA 94143, USA.*

E-mail: alatham30@gmail.com; leung.dennis@gene.com

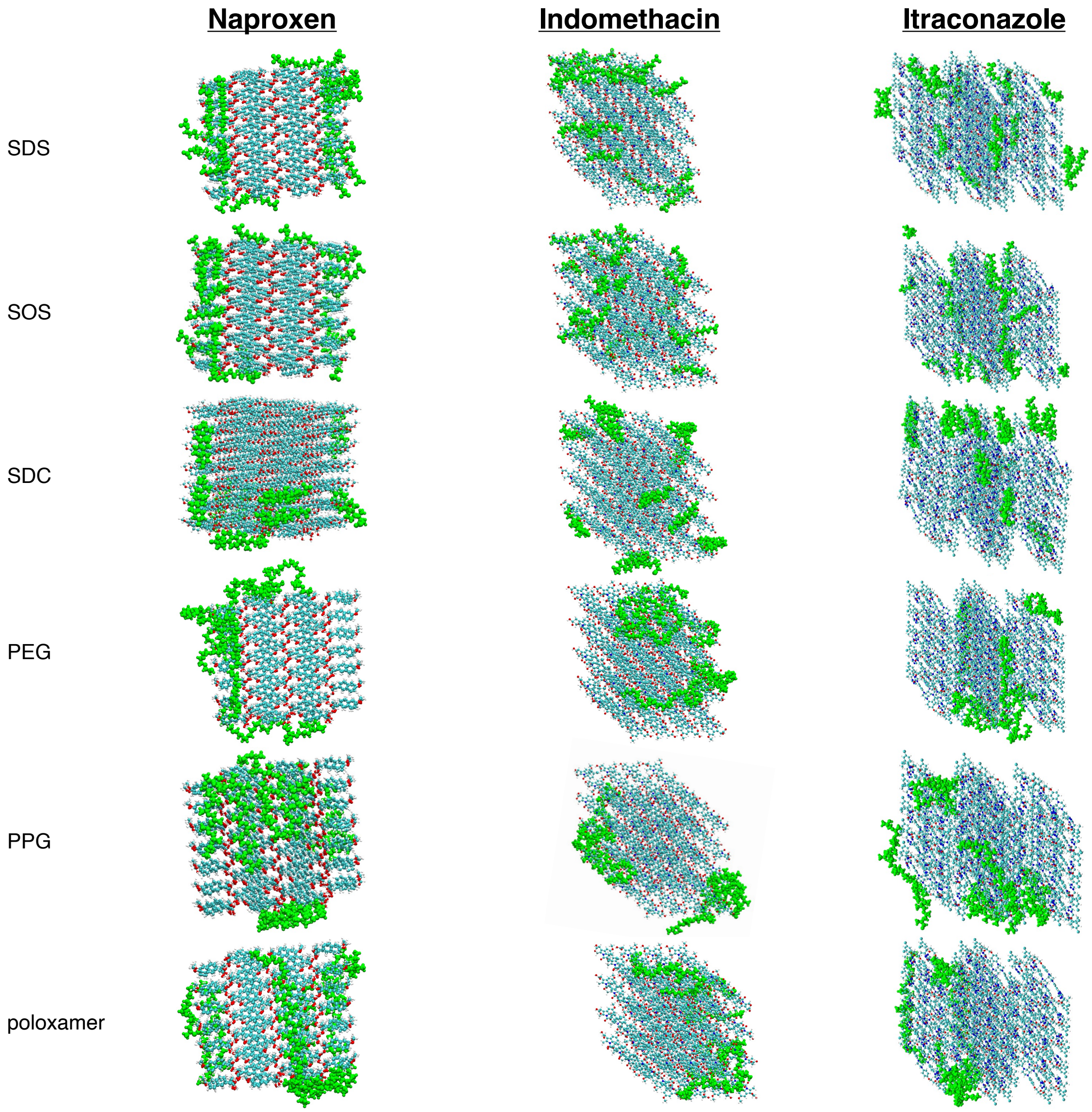

Figure S1: Images of the final configurations for all training set simulations. Each column corresponds to the same drug, while each row corresponds to the same excipient.

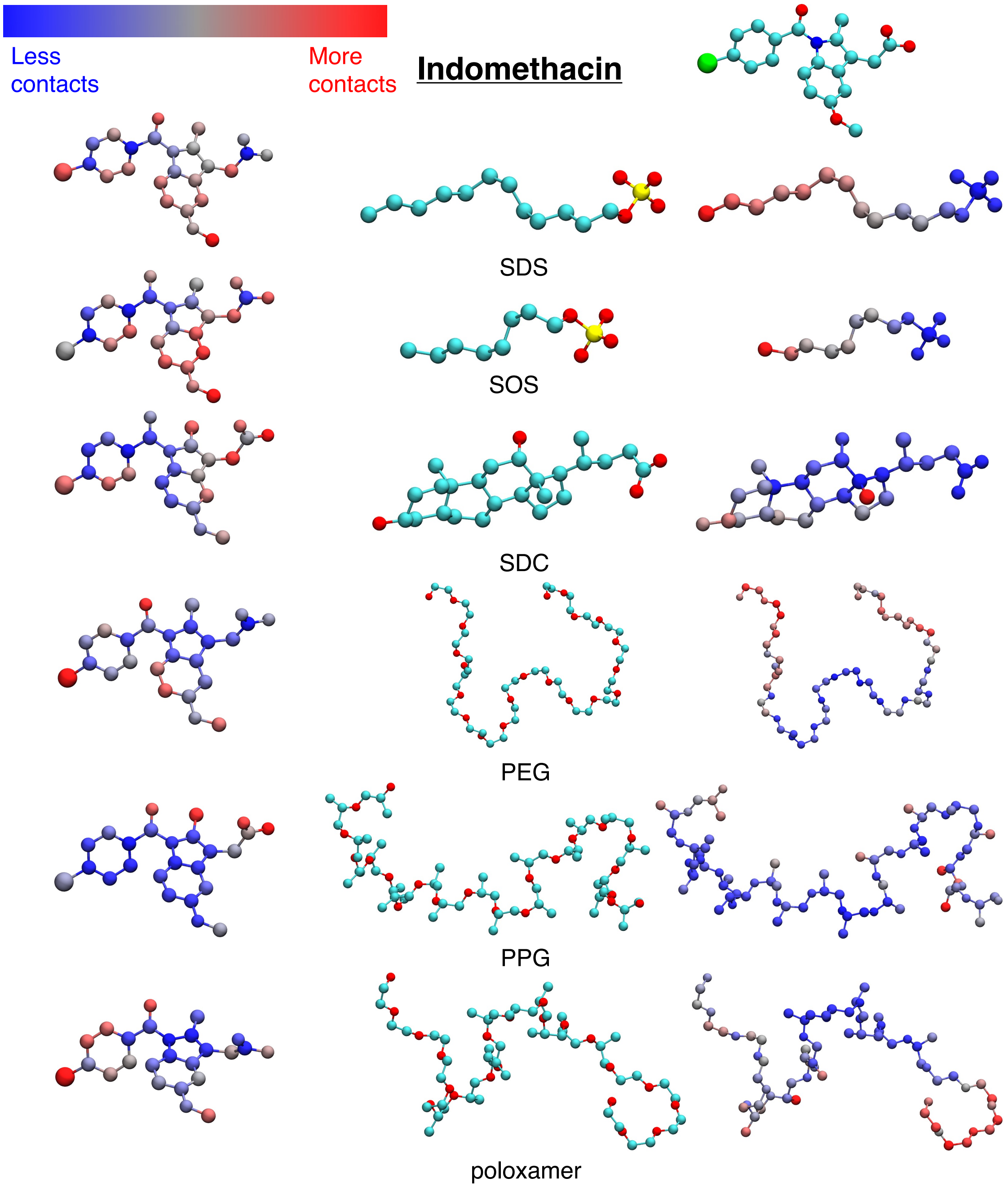

Figure S2: Heavy atom contact maps for where indomethacin molecules come in contact with excipient molecules. For each excipient, we show an indomethacin structure, colored by the contact map (left), a structure of the excipient, colored by atom type (middle), and a structure of the excipient, colored by the contact map (right). For structures colored by atom type, the color mapping is C–cyan, O–red, and S–yellow. For structures colored by the contact map, red regions represent where the drug and excipient are in contact the most, while blue regions represent where the drug and excipient are in contact the least.

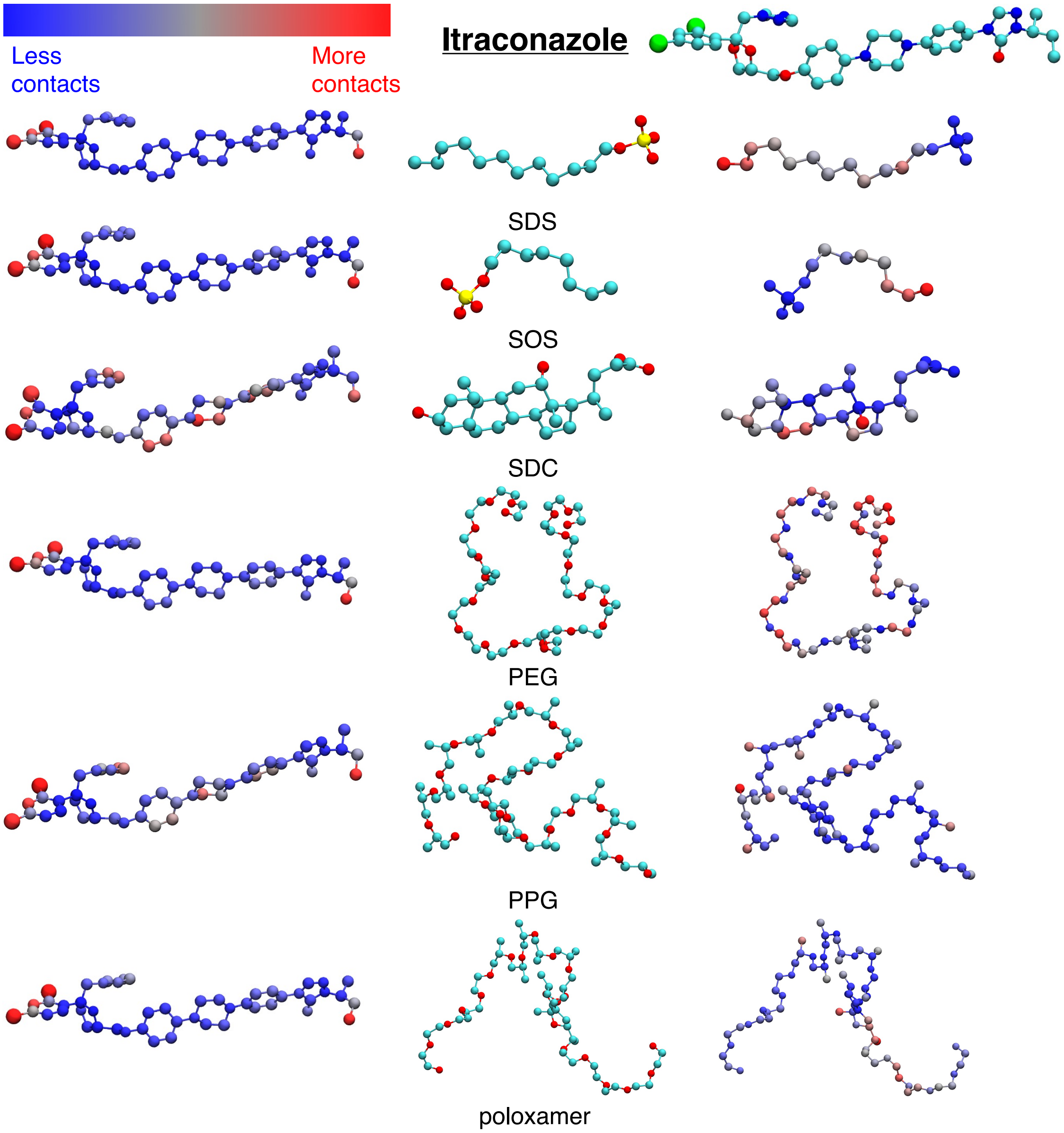

Figure S3: Heavy atom contact maps for where itraconazole molecules come in contact with excipient molecules. For each excipient, we show an itraconazole structure, colored by the contact map (left), a structure of the excipient, colored by atom type (middle), and a structure of the excipient, colored by the contact map (right). For structures colored by atom type, the color mapping is C–cyan, O–red, and S–yellow. For structures colored by the contact map, red regions represent where the drug and excipient are in contact the most, while blue regions represent where the drug and excipient are in contact the least.

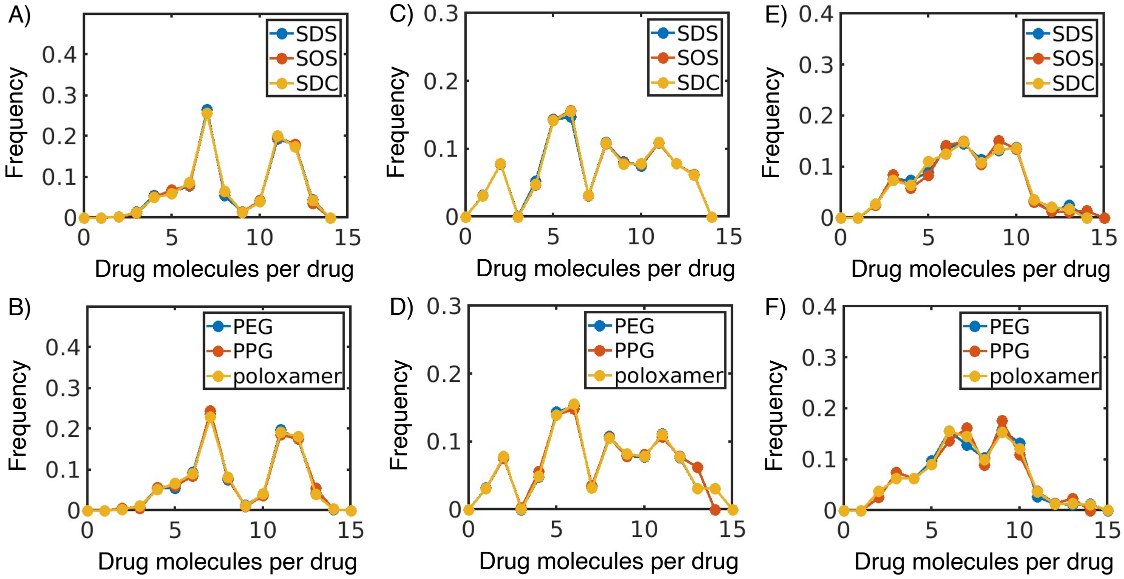

Figure S4: Frequency of the number of drug molecules bound to a single drug molecule during our simulations. Distributions are broken up between surfactant and polymer excipients for naproxen (A,B), indomethacin (C,D), and itraconazole (E,F). Due to restraints on the drug crystal, these plots are similar across different excipient combinations.

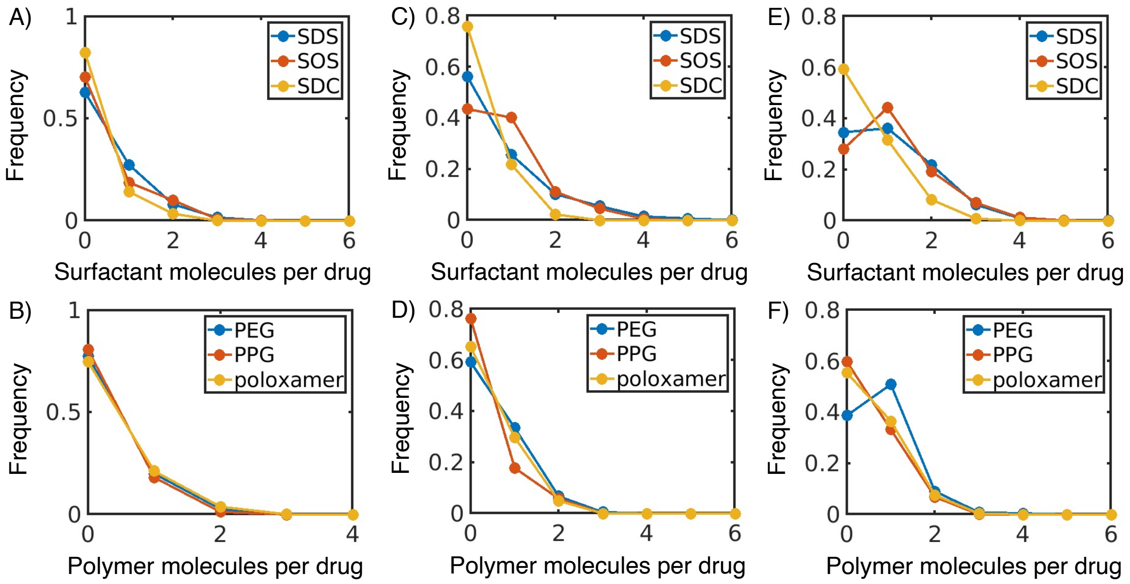

Figure S5: Frequency of the number of excipient molecules bound to a single drug molecule during our simulations. Distributions are broken up between surfactant and polymer excipients for naproxen (A,B), indomethacin (C,D), and itraconazole (E,F).

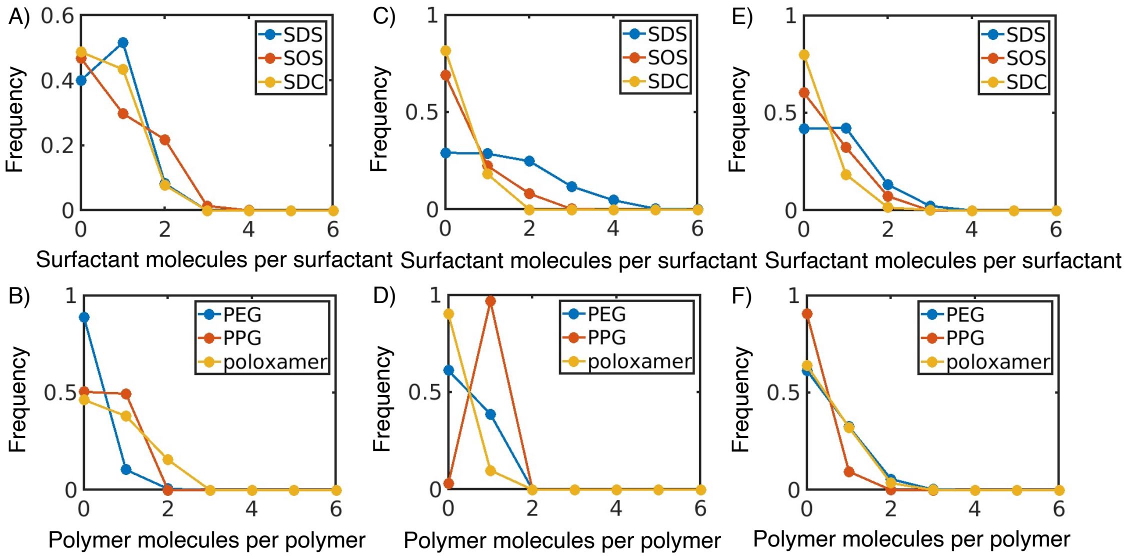

Figure S6: Frequency of the number of excipient molecules bound to a single excipient molecule during our simulations. Distributions are broken up between surfactant and polymer excipients for naproxen (A,B), indomethacin (C,D), and itraconazole (E,F).

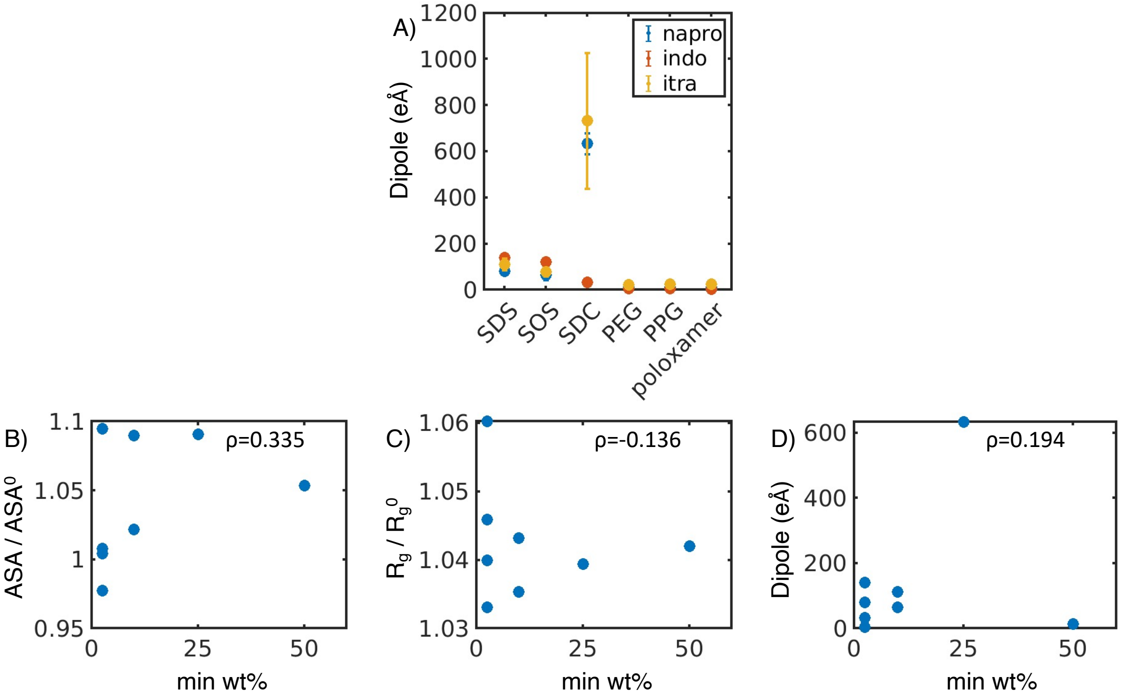

Figure S7: Other surface properties do not predict serve as good of predictors of nanoparticle stability as $FASA_p$. (A) The dipole moment for all drug-excipient combinations. Error bars represent the standard deviation of estimates from five independent time windows. (B) Accessible surface area of the excipient-drug system divided by the accessible surface area of the drug crystal without excipients (ASA/ASA^0), (C) radius of gyration of the excipient-drug system divided by the radius of gyration of the drug crystal without excipients (R_g/R_g^0), and (D) dipole moment are each plotted against the minimum weight percentage of excipient needed to form a stable nanosuspension (min wt%). The Pearson correlation coefficient (ρ) between the y-axis variable and min wt% is shown for each fit, and all are significantly lower than that of $FASA_p$ (Figure 4E).

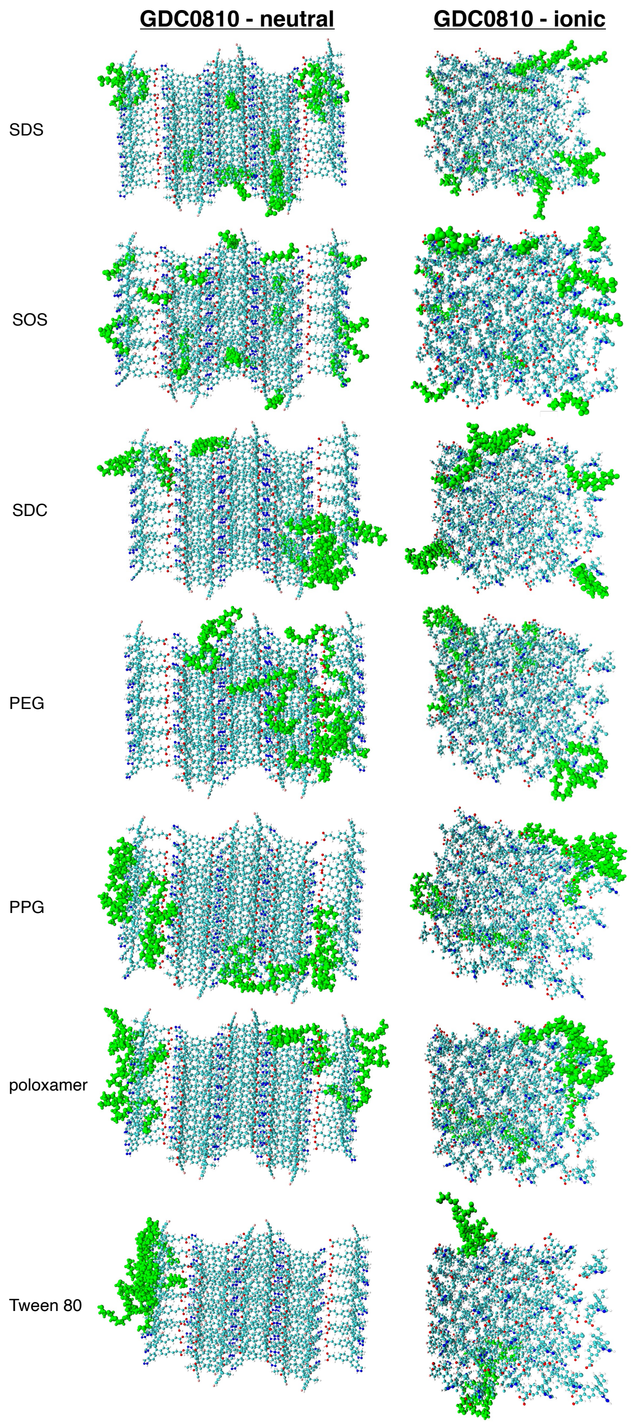

Figure S8: Images of the final configurations for all GDC-0810 simulations. Each column corresponds to either the neutral or charged GDC-0810 crystal, while each row corresponds to the same excipient.

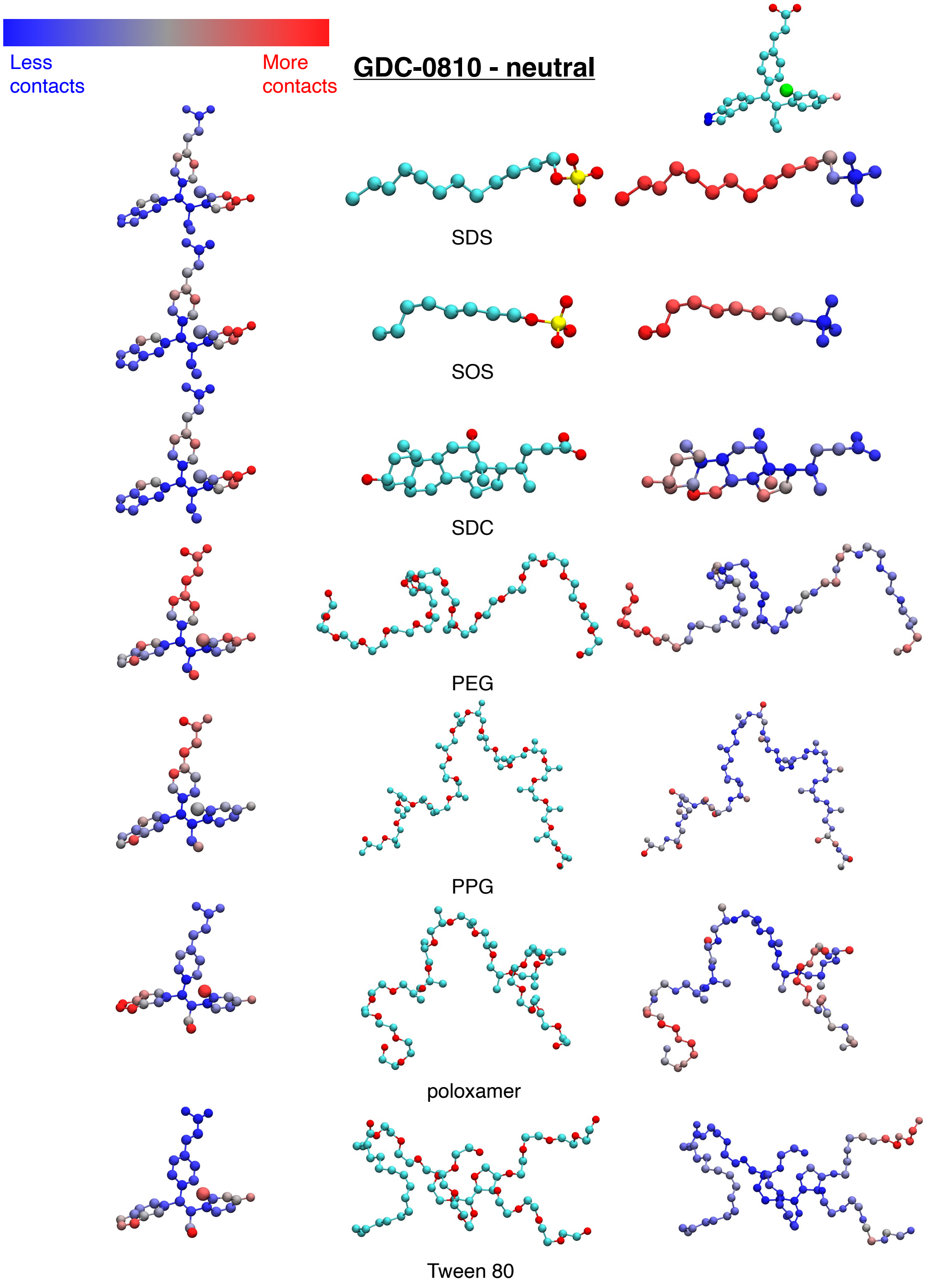

Figure S9: Heavy atom contact maps for where neutral GDC-0810 molecules come in contact with excipient molecules. For each excipient, we show a neutral GDC-0810 structure, colored by the contact map (left), a structure of the excipient, colored by atom type (middle), and a structure of the excipient, colored by the contact map (right). For structures colored by atom type, the color mapping is C–cyan, O–red, N–blue, F–pink, and Cl–green. For structures colored by the contact map, red regions represent where the drug and excipient are in contact the most, while blue regions represent where the drug and excipient are in contact the least.

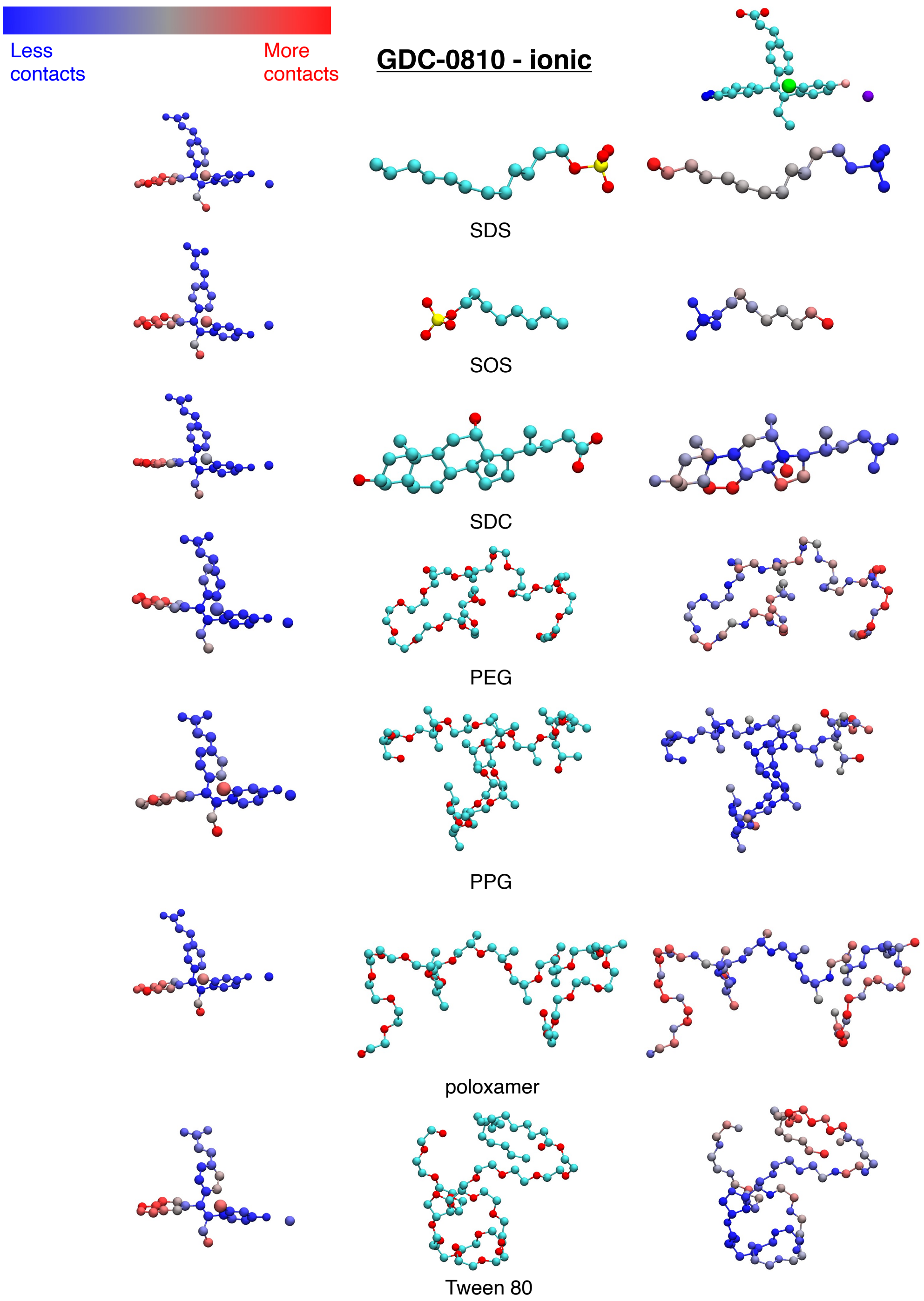

Figure S10: Heavy atom contact maps for where ionic GDC-0810 molecules come in contact with excipient molecules. For each excipient, we show an ionic GDC-0810 structure, colored by the contact map (left), a structure of the excipient, colored by atom type (middle), and a structure of the excipient, colored by the contact map (right). For structures colored by atom type, the color mapping is C–cyan, O–red, N–blue, F–pink, Cl–green, and K–purple. For structures colored by the contact map, red regions represent where the drug and excipient are in contact the most, while blue regions represent where the drug and excipient are in contact the least.

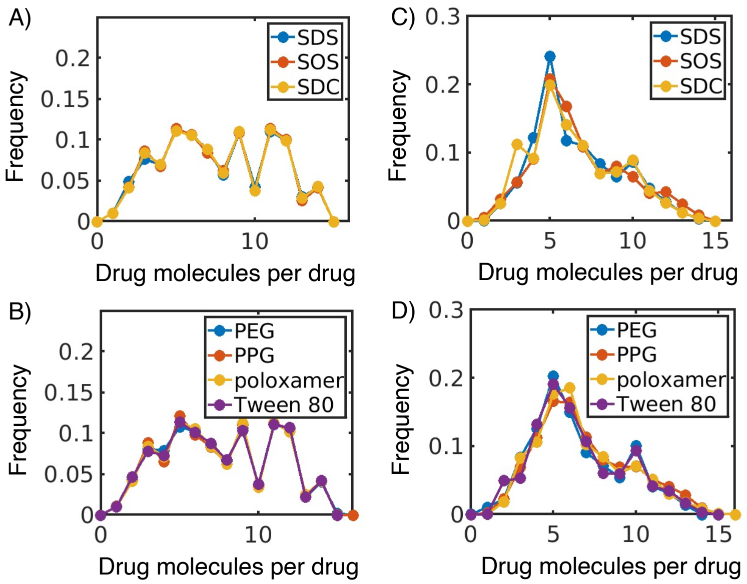

Figure S11: Frequency of the number of drug molecules bound to a single drug molecule during GDC-0810 simulations. Distributions are broken up between surfactant and polymer excipients for neutral GDC-0810 (A,B) and ionic GDC-0810 (C,D).

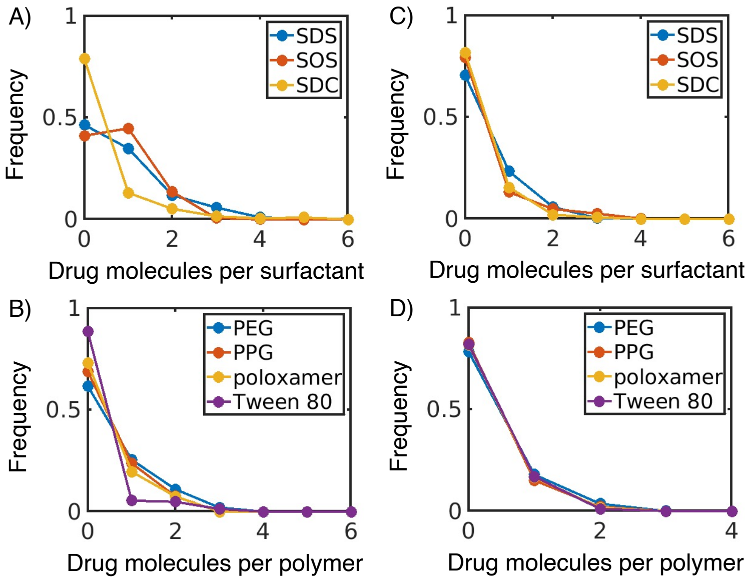

Figure S12: Frequency of the number of excipient molecules bound to a single drug molecule during GDC-0810 simulations. Distributions are broken up between surfactant and polymer excipients for neutral GDC-0810 (A,B) and ionic GDC-0810 (C,D).

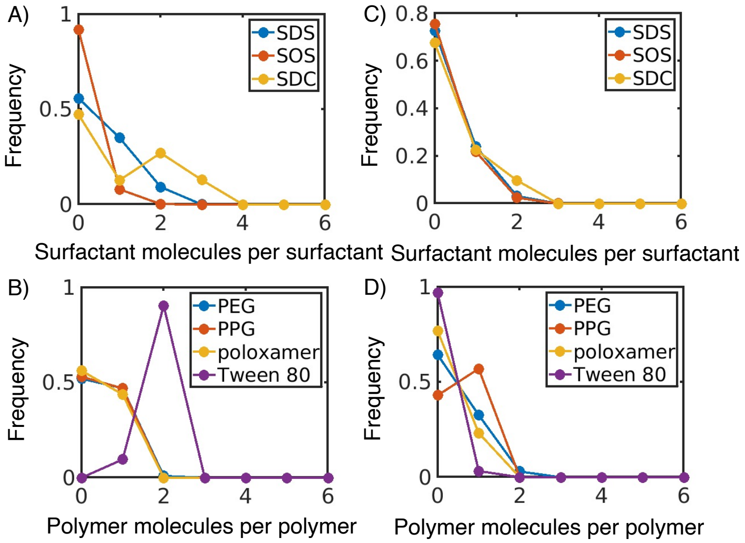

Figure S13: Frequency of the number of excipient molecules bound to a single excipient molecule during GDC-0810 simulations. Distributions are broken up between surfactant and polymer excipients for neutral GDC-0810 (A,B) and ionic GDC-0810 (C,D).

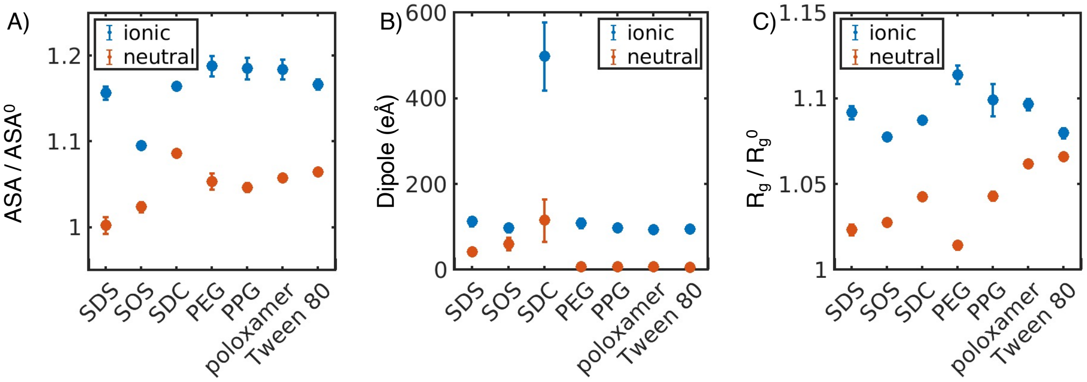

Figure S14: Additional surface properties for GDC-0180 crystals with various excipients. (A) Accessible surface area of the excipient-drug system divided by the accessible surface area of the drug crystal without excipients (ASA/ASA^0). (B) Dipole moment. (C) Radius of gyration of the excipient-drug system divided by the radius of gyration of the drug crystal without excipients (R_g/R_g^0). In each case, data is shown for the neutral (orange) and ionic (blue) crystal. Error bars represent the standard deviation of estimates from five independent time windows.

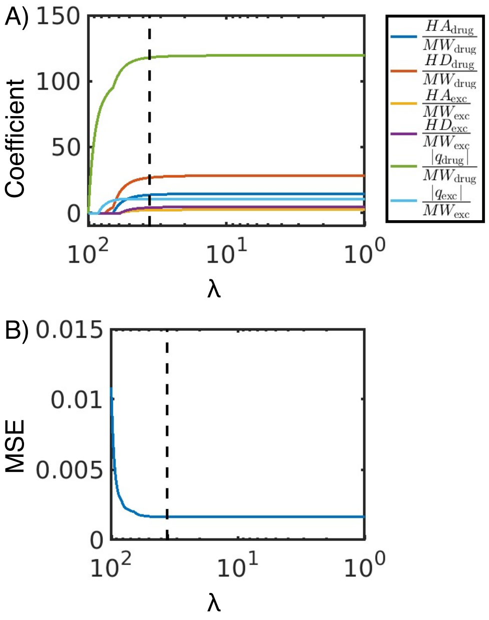

Figure S15: Fitting $\mathrm{FASA_p}$ from 2-D molecular properties with the least absolute shrinkage and selection operator (LASSO). (A) The dependence of linear fitting coefficient for each 2-D molecular property as a function of the regularization parameter (λ). The dashed line represents the optimal value calculated by 16-fold cross validation. (B) Mean-squared error (MSE) of cross validation as a function of λ. The optimal value of λ was chosen to minimize the MSE (black, dashed line).

Table S1: Additional experimental characterization of naproxen, indomethacin, and itraconazole nanosuspensions. For stable nanosuspensions, values represent the average particle diameter (nm), followed by the percent polydispersity (%Pd), reported as the polydispersity divided by the estimated hydrodynamic radius of the particle size population multiplied by 100. X represents no stable nanosuspension, while blank regions indicate that data was not taken for that combination of drug/excipient. This data utilizes the same methods as and complements the results of Ferrar et al.[?]

Drug	Excipient:drug (wt%)	PEG-200	PEG-400	poloxamer-188	SDC
		n=4-5	n=23	n=80, m=33	
naproxen	2.5		X		
	10.0		X		
	25.0		X		
	100.0		X		
	150.0		X		
	200.0	X	X		
indomethacin	2.5	X	X	380 / Multi	153 / Multi
	5.0				128 / Multi
	10.0	X	X	171 / Multi	107 / 28.9%
	25.0	X	X	115 / 25.2%	
	100.0	X	X	120 / 26.5%	
	150.0	X	X	144 / 26.1%	
itraconazole	10.0	X	X	X	X
	25.0	X	X	X	X
	100.0	X	X	X	X
	150.0	X	X		

Table S2: Minimum weight percentage of excipient: drug needed to form a stable nanosuspension (min wt%) based on experimental characterization in Table S1 and Ferrar et al.[?] Only drug-excipient combinations that resulted in a stable nanosuspension are listed.

Drug	Excipient	min wt%
naproxen	SDS	2.5
	SOS	10
	SDC	25
	poloxamer	50
indomethacin	SDS	2.5
	SDC	2.5
	poloxamer	2.5
itraconazole	SDS	10

Table S3: Summary of experimental results used for our logistic regression model. S denotes the drug-excipient combination resulted in a successful nanosuspension, F denotes the drug-excipient combination failed to create a stable nanosuspension, and X denotes that the drug-excipient combination was not tested experimentally.

Drug	SDS	SOS	SDC	PEG	PPG	poloxamer
naproxen	S	S	S	F	F	S
indomethacin	S	X	S	F	F	S
itraconazole	S	X	F	F	F	F

Table S4: Number of drug and excipient molecules used in creating our training set. N_{drug} is the number of drug molecules, N_{exc} is the number of excipient molecules, and Excipient:drug (wt%) is the weight ratio of all excipient molecules to all drug molecules.

Drug	Excipient	N_{drug}	N_{exc}	Excipient:drug (wt%)
naproxen	SDS	210	17	10.1
	SOS	210	21	10.1
	SDC	210	12	10.3
	PEG	210	5	9.3
	PPG	210	4	9.7
	poloxamer	210	5	10.7
indomethacin	SDS	128	16	10.1
	SOS	128	20	10.1
	SDC	128	11	10.0
	PEG	128	5	9.8
	PPG	128	4	10.3
	poloxamer	128	4	9.1
itraconazole	SDS	80	20	10.2
	SOS	80	24	9.9
	SDC	80	14	10.3
	PEG	80	6	9.6
	PPG	80	5	10.4
	poloxamer	80	5	9.2

Table S5: Number of drug and excipient molecules used in creating our test set. N_{drug} is the number of drug molecules, N_{exc} is the number of excipient molecules, and Excipient:drug (wt%) is the weight ratio of all excipient molecules to all drug molecules. GDC-0810 is assumed to be neutral for mass calculations.

Drug	Excipient	N_{drug}	N_{exc}	Excipient:drug (wt%)
GDC-0810	SDS	96	15	10.1
	SOS	96	18	9.7
	SDC	96	10	9.7
	PEG	96	5	10.5
	PPG	96	4	11.0
	poloxamer	96	4	9.7
	Tween 80	96	3	9.2

Table S6: 2-D properties of drug molecules, used for fitting in Figure 8. MW_{drug} is the molecular weight of the drug molecule, HA_{drug} is the number of hydrogen bond acceptors in the drug molecule, HD_{drug} is the number of hydrogen bond donors in the drug molecule, and q_{drug} is the charge of each drug molecule. MW_{drug}, HA_{drug}, and HD_{drug} are calculated through the chemalot open source package.[?]

Drug	MW_{drug}	HA_{drug}	HD_{drug}	q_{drug}
naproxen	230.26	3	1	0
indomethacin	357.79	5	1	0
itraconazole	705.63	12	0	0
GDC-0810 - neutral	446.90	4	2	0
GDC-0810 - ionic	446.90	5	1	-1

Table S7: 2-D properties of excipient molecules, used for fitting in Figure 8. MW_{exc} is the molecular weight of the excipient molecule, HA_{exc} is the number of hydrogen bond acceptors in the excipient molecule, HD_{exc} is the number of hydrogen bond donors in the excipient molecule, and q_{exc} is the charge of each excipient molecule. MW_{exc}, HA_{exc}, and HD_{exc} are calculated through the chemalot open source package.[?]

Excipient	MW_{exc}	HA_{exc}	HD_{exc}	q_{exc}
SDS	266.40	4	1	-1
SOS	210.29	4	1	-1
SDC	392.57	4	3	-1
PEG	899.07	21	2	0
PPG	1179.60	21	2	0
poloxamer	1039.33	21	2	0
Tween 80	1133.44	22	3	0
